# Responses in gut microbiota and fat metabolism to a halogenated methane analogue in Sprague Dawley rats

**DOI:** 10.1111/1751-7915.12256

**Published:** 2015-03-06

**Authors:** Yong Su, Yu-Heng Luo, Ling-Li Zhang, Hauke Smidt, Wei-Yun Zhu

**Affiliations:** 1 Laboratory of Gastrointestinal Microbiology, Nanjing Agricultural UniversityNanjing, 210095, China; 2 Laboratory of Microbiology, Agrotechnology and Food Sciences Group, Wageningen UniversityWageningen, 6703 HB, The Netherlands

## Abstract

Recent studies on germ-free mice show that intestinal methanogens may be closely associated with host's adipose metabolism. The present study aimed to investigate effects of inhibition of intestinal methanogen populations on host fat metabolism by establishing a healthy Sprague Dawley (SD) rat model through the intragastric administration of bromochlordomethane (BCM). Forty-five 8-week old healthy male SD rats were randomly divided into five groups including one control and four BCM treatments. The experiment lasted 60 days with two separate 30-day experimental periods. At the end of first period, three BCM treatment groups were further used: one group continued with BCM treatment, one group stopped with BCM treatment, and the other one inoculated with faecal mixture of methanogens from rats. Results showed that the methanogen population in feces was reduced sixfold with no effect on the bacterial community by daily dosing with BCM. Daily gain, epididymal fat pad weight, levels of plasma low-density lipoprotein and cholesterol were significantly higher in the BCM-treated animals, while the high-density lipoprotein was lower than that of the control. The expression of *PPARγ*, *LPL*, *PP2A*, *SREBP-1c*, *ChREBP*, *FASN* and *adiponectin* genes in BCM treatment group was universally upregulated, while the expression of *Fiaf* gene was downregulated. After termination of BCM treatment and followed either with or without re-inocubation with faecal methanogen mixture, the rats had their faecal methanogen populations, blood parameters and gene expression returned to the original level. Results suggest that regulation of gut methanogens might be a possible approach to control host body weight.

## Introduction

Recent research has shown that there is a close relationship between the gut microbiota and host's energy metabolism and adipogenesis in monogastric animals including humans (Ley *et al*., 2005). A series of studies on germ-free mice illustrated that intestinal bacteria such as *Bacteroides*, *Clostridium* and other groups in phylum Firmicutes enriched on glycometabolism associated pathway and played an essential role on host's energy absorption and lipid metabolism (Ley *et al*., 2005; Turnbaugh *et al*., 2006). Researchers also found that the colonization of gnotobiotic mice with *Bacteroides thetaiotaomicron* and *Methanobrevibacter smithii* increased their population density in the distal gut (Samuel and Gordon, 2006). The colonization of *M. smithii* can improve the ability of *B. thetaiotaomicron* to degrade polyfructose-containning glycans and enhance the ability of the host to harvest and store calories from diet, while another hydrogen remover *Desulfovibrio piger* shows no such function (Samuel and Gordon, 2006). All these studies, however, were conducted with a single or a few pure microbial species. A healthy conventional animal harbours complex diverse methanogen populations. Therefore, it would be interesting to understand the effect of gut methanogens as a population on the host's growth and health including fat metabolism.

Pigs, with complex methanogens in their gut (Mao *et al*., 2011; Luo *et al*., 2012), were estimated to typically lose 1.2% of ingested energy as methane (Monteny *et al*., 2001). Our previous study by comparison between lean pigs and obese pigs, indicated that the Landrace pig (lean) harboured a greater diversity and higher numbers of methanogen *mcrA* gene copies than the Erhualian pig (obese) (Luo *et al*., 2012). Thus, the difference in methanogen abundance in the gut may be related to the fatness or leanness in these two pig breeds, which may further link to the fat metabolism in pigs or even humans. However, no information is available on the relation of gut methanogen populations and the growth or metabolism of the host.

Bromochloromethane (BCM), as a specific inhibitor of methanogens, is believed to react with reduced vitamin B12 and results in the inhibition of cobamide-dependent methyl group transfer in methanogenesis (Goel *et al*., 2009). Bromochloromethane has previously been used to reduce ruminal methane production without adversely affecting the animal performance (Denman *et al*., 2007). However, our preliminary study showed that BCM administration of C57BL/6J mice through drinking water failed to inhibit the methanogen populations in the cecum (Ma *et al*., 2012). In the present study, BCM was further used to inhibit gut methanogens in a Sprague Dawley (SD) rat model by intragastric administration, to investigate the effects of BCM on gut microbial ecology and fat metabolism of the host, with the attempt to gain information on the link between gut methanogen populations and fat metabolism in a healthy conventional host.

## Results

### Effects of BCM on the number of mcrA gene copies and bacterial community in the feces of rats

**Fig 1:**
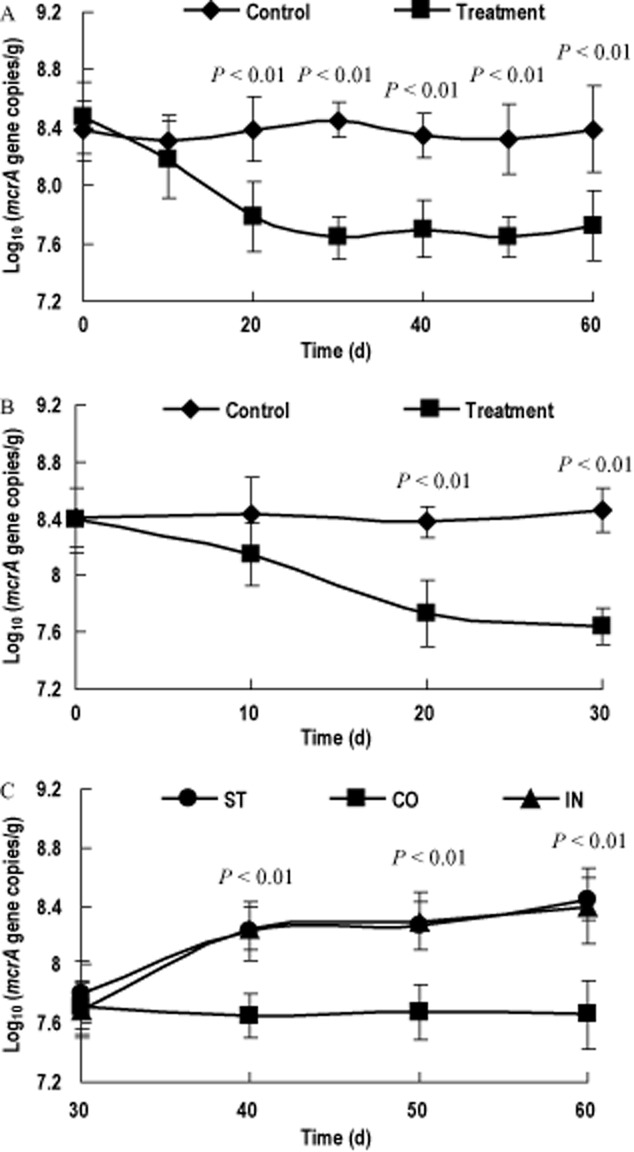
Copy numbers of *mcr*A gene in the feces of rats (A: control and treatment groups during the pre-experiment period; B: control and treatment groups during the first experimental period; C: group continued with BCM treatment (CO), group stopped with BCM treatment (ST) and group inoculated with faecal mixture of methanogens from healthy rats (IN) during the second experimental period). For each experimental time, *P* value was added when significant difference was observed among different groups.

The results of pre-experiment showed that after the first 30-day BCM treatment, the number of intestinal methanogens was significantly reduced by BCM treatment with time in the first 30 days (*P* < 0.01), then remained stable in the following 30 days (Fig. 1A). Therefore, we selected 30-day as one experimental period during the formal experiment. As shown in Fig. 1B, similar to the pre-experiment, at the end of first experimental period, the faecal methanogen population was reduced sixfold with BCM treatment (*P* < 0.01). However, there was a significant increase on the amount of methanogens in ST (stopped with BCM treatment) and IN (stopped with BCM treating and inoculated with faecal methanogen mix from healthy rat) groups as compared with that in CO (continued with BCM treatment) group during the second experimental period (Fig. 1C).

**Fig 2:**
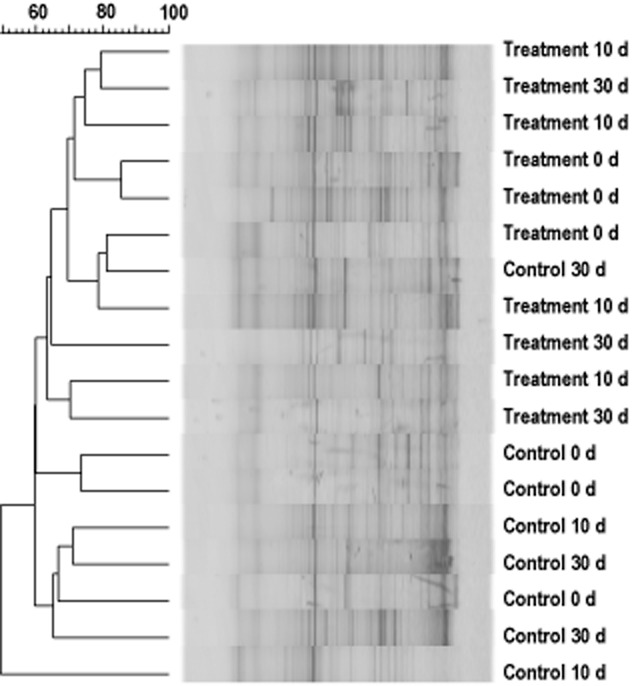
Similarity analysis of DGGE profiles of the bacterial community in feces of rats in control and BCM treatment groups.

To investigate whether BCM affect the faecal bacteria, the bacterial community diversity and abundances of the major bacterial groups were determined. Similarity analysis of denaturing gradient gel electrophoresis (DGGE) profiles showed that both factors BCM and experimental time failed to separate the samples to the same clusters (Fig. 2). In addition, real-time polymerase chain reaction (PCR) assays showed that there were no significant difference in the 16S rRNA gene copies of total bacteria, Firmicutes and Bacteroidetes between the control and the treatment groups at the end of the first experimental period (Table S1).

### Effects of BCM on rat growth characteristics and biochemical parameters in blood serum

As shown in Table 1, during the first experimental period, the daily gain and the weight of epididymal fat pad of rats in the treatment group were significantly higher than those in the control group (*P* < 0.01). Bromochlordomethane treatment did not affect glucose and triglyceride levels in the blood serum of rats. As compared with the control group, the level of high-density lipoprotein (HDL) in BCM treatment group was significantly higher, while low-density lipoprotein (LDL) and cholesterol levels were significantly lower (*P* < 0.01). During the second experimental period, both daily gain and the weight of epididymal fat pad of the rats in ST and IN groups were significantly lower than those in the CO group (*P* < 0.05) (Table 2). Lower levels of LDL and cholesterol in rat blood serum in the ST and IN groups were found as compared with those in the CO group (*P* < 0.05). There were no significant differences in glucose and triglyceride levels in the blood serum of rats among the three groups. No differences in all parameters were found between ST and IN groups. During the whole experiment, the feed intake of rats was not affected by the BCM treatment.

**Table 1:** Physiological and biochemical parameters of rats in the control and treatment groups

Parameters	Groups	
	Control	Treatment
HDL (mmol/l)	1.11 ± 0.13	0.78 ± 0.13**
LDL (mmol/l)	0.54 ± 0.07	0.74 ± 0.11**
Cholesterol (mmol/l)	1.36 ± 0.26	1.72 ± 0.11**
Glucose (mmol/l)	3.01 ± 0.43	3.28 ± 0.50
Triglyceride (mmol/l)	1.36 ± 0.55	1.88 ± 0.34
Epididymal fat pad (g)	3.94 ± 0.15	6.46 ± 0.50**
ADG (g/d)	4.49 ± 0.46	5.68 ± 0.42**

** *P* < 0.01.

Data were analysed with Student's *t*-test, and confidence interval is 95% (*n* = 9).

ADG, average daily gain; EFP, weight of epididymal fat pad.

**Table 2:** Physiological and biochemical parameters of rats in the CO, ST and IN groups

Parameter	Groups		
	CO	ST	IN
HDL (mmol/l)	0.91 ± 0.07^a^	0.99 ± 0.11^ab^	1.02 ± 0.06^b^
LDL (mmol/l)	0.708 ± 0.135^a^	0.494 ± 0.088^b^	0.454 ± 0.06^b^
Cholesterol (mmol/l)	1.566 ± 0.183^a^	1.24 ± 0.15^b^	1.278 ± 0.14^b^
Glucose (mmol/l)	3.722 ± 0.233	3.63 ± 0.28	3.556 ± 0.46
Triglyceride (mmol/l)	1.403 ± 0.328	1.16 ± 0.23	1.141 ± 0.55
EFP (g)	7.850 ± 0.327^a^	7.24 ± 0.25^b^	7.229 ± 0.333^b^
ADG (g/d)	2.952 ± 1.162^a^	1.53 ± 0.55^b^	1.256 ± 0.46^b^

Data were analysed with ANOVA, and confidence interval is 95% (*n* = 9). The variant alphabetical superscript in the same row means significant difference at *P* < 0.05.

ADG, average daily gain; EFP, weight of epididymal fat pad.

### Effects of BCM on expression of fat metabolism-related genes in the epididymal fat pad, liver and colon

The expression of nine lipid metabolism associated genes (*PPARγ*, *Fiaf*, *leptin*, *LPL*, *PP2A*, *SREBP-1c*, *ChREBP*, *adiponectin* and *FASN*) in the epididymal fat pad, liver and colon of rats was measured using relative real-time PCR. Three housekeeping genes (*β-actin*, *HPRT*, and *GAPDH*) were selected as reference. During the first experimental period, as compared with the control, gene expression of *PPARγ*, *LPL*, *PP2A*, *SREBP-1c*, *ChREBP*, *FASN* and *adiponectin* was upregulated in the three tissues of rats treated with BCM, while there was a contrary change for the expression of *Fiaf* gene (Table 3). Bromochloromethane treatment didn't affect the expression of *leptin* gene. During the second experimental periods, as compared with the CO group, the expression of *Fiaf* gene in ST and IN groups were upregulated, while expression of *PPARγ*, *LPL*, *PP2A*, *SREBP-1c*, *ChREBP* and *FASN* genes was downregulated (Table 4). The expression of *leptin* gene in IN group was upregulated. During the whole experiment, the expression of *adiponectin* in the epididymal fat pad of rats was upregulated by BCM treatment and recovered once BCM dosing was terminated, while it was not detected in the liver and colon of rats.

**Table 3:** Relative expression of adipose metabolism associated genes in the epididymal fat pad, liver and colon of rats (treatment versus control)

Genes	Epididymal fat pad		Liver		Colon	
	Control	Treatment	Control	Treatment	Control	Treatment
*PPARγ*	1.02 ± 0.26	7.74 ± 1.64**	1.01 ± 0.17	5.49 ± 1.33**	1.01 ± 0.17	2.53 ± 0.51**
*Fiaf*	1.02 ± 0.23	0.56 ± 0.02*	1.03 ± 0.27	0.39 ± 0.07*	1.06 ± 0.42	0.90 ± 0.06
*LPL*	1.00 ± 0.08	4.15 ± 0.57**	1.04 ± 0.20	4.75 ± 0.30**	1.01 ± 0.18	2.91 ± 0.71*
*PP2A*	1.03 ± 0.03	2.37 ± 0.52*	1.01 ± 0.13	2.46 ± 0.18**	1.02 ± 0.24	2.51 ± 0.23*
*SREBP-1c*	1.01 ± 0.06	6.56 ± 0.41**	1.00 ± 0.10	3.76 ± 0.57**	1.01 ± 0.15	2.28 ± 0.28*
*ChREBP*	1.03 ± 0.05	3.27 ± 0.21**	1.01 ± 0.09	4.01 ± 0.50**	1.02 ± 0.18	4.71 ± 0.65**
*FASN*	1.01 ± 0.09	3.29 ± 0.50**	1.01 ± 0.13	3.77 ± 0.26**	1.02 ± 0.19	7.73 ± 1.19**
*Leptin*	1.01 ± 0.07	0.92 ± 0.28	1.08 ± 0.45	0.48 ± 0.18	1.03 ± 0.27	1.21 ± 0.11
*Adiponectin*	1.02 ± 0.13	9.35 ± 1.79**	–	–	–	–

* *P* < 0.05 (compared with the control, confidence interval is 95%)

** *P* < 0.01 (compared with the control, confidence interval is 95%).

*Fiaf*, fasting-induced adipose factor; *LPL*, lipoprotein lipase; *PP2A*, protein phosphatase 2A; –, no expression detected

**Table 4:** The relative expressions (folds) of adipose associated genes in the epididymal fat pad, liver and colon of rats

Genes	Epididymal fat pad			Liver			Colon		
	CO	ST	IN	CO	ST	IN	CO	ST	IN
*PPARγ*	1.00 ± 0.03^a^	0.15 ± 0.04^c^	0.26 ± 0.04^b^	1.02 ± 0.19^a^	0.26 ± 0.05^b^	0.27 ± 0.06^b^	1.01 ± 0.15^a^	0.39 ± 0.01^b^	0.39 ± 0.06^b^
*Fiaf*	1.01 ± 0.07^b^	5.14 ± 0.70^a^	5.65 ± 0.75^a^	1.04 ± 0.35^c^	2.43 ± 0.38^b^	3.39 ± 0.12^a^	1.01 ± 0.19^b^	2.19 ± 0.53^a^	2.76 ± 0.23^a^
*LPL*	1.00 ± 0.15^a^	0.25 ± 0.03^b^	0.33 ± 0.01^b^	1.00 ± 0.12^a^	0.23 ± 0.01^b^	0.19 ± 0.01^b^	1.02 ± 0.23^a^	0.41 ± 0.10^b^	0.40 ± 0.07^b^
*PP2A*	1.02 ± 0.21	0.90 ± 0.16	1.09 ± 0.16	1.01 ± 0.13^a^	0.57 ± 0.03^b^	0.58 ± 0.04^b^	1.02 ± 0.24^ab^	0.74 ± 0.06^b^	1.22 ± 0.14^a^
*SREBP-1c*	1.02 ± 0.12^a^	0.12 ± 0.01^b^	0.13 ± 0.02^b^	1.01 ± 0.21^a^	0.41 ± 0.03^b^	0.30 ± 0.05^b^	1.00 ± 0.03^a^	0.20 ± 0.03^b^	0.26 ± 0.04^b^
*ChREBP*	1.01 ± 0.15^a^	0.35 ± 0.05^b^	0.33 ± 0.06^b^	1.00 ± 0.10^a^	0.41 ± 0.01^b^	0.37 ± 0.05^b^	1.00 ± 0.07^a^	0.14 ± 0.031^b^	0.20 ± 0.03^b^
*FASN*	1.00 ± 0.06^a^	0.18 ± 0.02c	0.32 ± 0.04^b^	1.01 ± 0.13^a^	0.37 ± 0.01^b^	0.27 ± 0.04^b^	1.02 ± 0.26^a^	0.14 ± 0.02^b^	0.16 ± 0.05^b^
*Leptin*	1.02 ± 0.24^b^	1.67 ± 0.60^b^	3.94 ± 1.47^a^	1.01 ± 0.17^b^	4.35 ± 0.40^a^	4.81 ± 0.58^a^	1.02 ± 0.23^b^	0.93 ± 0.16^b^	1.67 ± 0.31^a^
*Adiponectin*	1.00 ± 0.12^a^	0.09 ± 0.03^b^	0.08 ± 0.01^b^	–	–	–	–	–	–

The variant alphabetical superscript in the same row from the same tissue means significant difference at *P* < 0.05.

*Fiaf*, fasting-induced adipose factor; *LPL*, lipoprotein lipase; *PP2A*, protein phosphatase 2A; –, no expression detected.

## Discussion

In a number of compounds which have been found to reduce ruminal methane production, BCM has been widely utilized for its high efficiency on control methane emission and relative safety to animal body (Trei *et al*., 1972). Bromochloromethane dosed at 0.3 and 0.6 g/100 kg life weight could significantly reduce methane production without affecting dry matter intake (Tomkins and Hunter, 2004). The inhibition of methanogens by BCM was also confirmed by *in vitro* and *in vivo* studies in goats and cattle (Denman *et al*., 2007; Goel *et al*., 2009; Abecia *et al*., 2012). In monogastric animals, our preliminary study with mice showed that BCM administration of C57BL/6J mice through drinking water failed to inhibit the methanogen populations in the cecum based on the quantification of methanogenic 16S rRNA gene (Ma *et al*., 2012). This was probably due to low dosage the mice received or the inaccurate method used for determination of methanogen numbers. Breath methane production can be measured to indicate the activation of methanogenes *in vivo*. Nevertheless, it is hard to quantify the methane production in rat *in vivo*. Whereas, methyl coenzyme-M reductase subunit A (mcrA) gene has been reported as a functional gene during methanogenesis of archaea (Hallam *et al*., 2003), and thus, it is recognized that the expression of *mcrA* could be used as a measurement for the activation of methanogens. In the present study, by determination of *mcrA* gene copies, we successfully constructed a gut methanogen inhibition rat model by intragastric administration of BCM. During the pre-experiment in our study, we found that after 30-day BCM treating, the number of intestinal methanogens was decreased significantly and remained constant in the following 30 days. After termination of BCM treatment, a significant recover of colonic methanogen population was observed. In addition, dissociation curve analysis of *mcrA* amplicons showed a main peak at the melting temperatures of 82°C for all samples and the standard strain *M. smithii* (data no shown). We also found a decrease in the intensity of the *Methanobrevibacter* spp. specific peak in BCM treatment group compared with the control group, which is similar to the previous finding in the rumen of cattle (Denman *et al*., 2007).

Firmicutes and Bacteroidetes are regarded as the main bacterial groups in the human and some monogastric animals such as rat, mouse and pig, which consist of about 90% of all phylogenetic types (Ley *et al*., 2006). Bromochloromethane treatment did not affect the numbers of total bacteria and these two phyla. Clustering of similarly of DGGE profiles also failed to separate samples from different groups, which suggests that BCM treatment did not affect the general bacterial community in the gut of rats. This finding is consistent with the results of researches on the ruminant where BCM treatment only affected the diversity of methanogens rather than the bacterial community based on the real-time PCR assay (Denman *et al*., 2007). However, a recent study found that BCM could affect the diversity of acetogens (another H_2_ utilizing bacterial group) in the bovine rumen, and change in acetogenic community structure in response to methane inhibition (Mitsumori, *et al*., 2014). As DGGE and real-time PCR can only analyse the predominant or known microbial groups, high-throughput techniques are needed to analyse microbiota in future studies. Furthermore, changes in the microbial community can influence the metabolites (e.g. short chain fatty acid) concentrations in the gut. To further understand the regulation mechanism of methanogen inhibition on host metabolism, it may be also necessary to investigate microbial metabolic profiles using metabolomic analysis.

Nevertheless, we found that coupled with the inhibition of gut methanogens, blood parameters were affected after BCM treatment. The levels of serum LDL and cholesterol were significantly increased compared with control while the level of HDL decreased. Daily gain and the weight of epididymal fat pad of treatment were also markedly elevated. With the re-colonization of gut methanogens, the average daily gain and weight of epididymal fat pad were statistically lower compared with continued BCM treatment, and there was a consistent change on the blood serum parameters. Research has demonstrated that high levels of serum glucose, triglyceride (TG), LDL and cholesterol are always involved in obesity-associated diseases such as type 2 diabetes, hypertension and some other chronic diseases (Xu *et al*., 2010). In the present study, methanogens-inhibited rats showed an obese trend as compared with the normal rats. However, it seems that this effect was gradually alleviated by the re-colonization and the recovery of the disappeared methanogens. As mentioned above, methane production is recognized as a waste of digestible energy (Tomkins and Hunter, 2004; Denman *et al*., 2007). Thus, the inhibition of methanogens may result in the absorption of additional energy which otherwise released as gas. It was also found that there was a slightly increase on average daily gain after treating with BCM in steer (Tomkins and Hunter, 2004), which is in agreement with our result.

In addition to the physiological and biochemical parameters, the expressions of several obesity-associated genes in colon were determined. Interestingly, we found that after treatment with BCM, except *Fiaf* gene, most of detected genes were upregulated. Peroxisome proliferator-activated receptor γ (PPARγ) is one of the members of the family of orphan nuclear receptors that function as transactivators of fat-specific genes and thus are dominant activators of fat cell differentiation (Dubuquoy *et al*., 2002). *Fiaf*, one of the target genes of *PPARγ* in white adipose tissues, is principally involved in lipid metabolism and secreted by adipocytes, liver and enterocyte (Rawls *et al*., 2004; Kersten, 2005). *Fiaf* is also proved as a direct regulator-mediating energy metabolism of hot and gut microbes (Bäckhed *et al*., 2004). In the current study, we found that the expression of *PPARγ* gene was upregulated, and *Fiaf* gene was both downregulated after the administration of BCM. The level of *LPL* gene expression was also inordinately upregulated. With the recolonization of colonic methanogens, the expression of *Fiaf* was upregulated, while *PPARγ* and *LPL* were downregulated compared with those continued with BCM treatment. Similarly, previous study also found that the expression of *PPARγ* in post-obese rats was clearly upregulated (Milan *et al*., 2002). Although it needs further elucidation on the mode of action of BCM, our present study demonstrated that the inhibition of methanogens in gut by BCM was coupled with the increased expression of *PPARγ*, which may sequentially decrease the expression of *Fiaf* gene, then enhance the level of *LPL*.

Fatty acid synthase (FASN) is necessary for *de novo* synthesis of long-chain, saturated fatty acid from acetyl coenzyme A (CoA), manlonyl CoA and Nicotinamide adenine dinucleotide phosphate (NADPH) (Wang *et al*., 2004). It is reported that the stimulation of FASN activity and the expression of *FASN* gene can induce the increase of body fat through regulating metabolic consequences of caloric excess such as insulin resistance, dyslipidaemia and altered adipokine serum profile (Berndt *et al*., 2007). It is also known that *SREBP-1c* and *ChREBP* can stimulate lipogenesis-associated genes including *FASN* (Weickert and Pfeiffer, 2006). Sterol regulatory element-binding protein-1c (SREBP-1c) and carbohydrate response element-binding protein (ChREBP) can mediate hepatocyte lipogenic response to insulin and glucose and appear to act synergistically (Dridi *et al*., 2005). Conventionalization of germ-free mice could increase liver ChREBP messenger ribonucleic acid (mRNA), and to a lesser extent, SREBP-1c mRNA levels (Bäckhed *et al*., 2004). In agreement with these findings, our results showed that compared with control, rats with BCM treatment clearly showed heavier epididymal fat pad and daily weight gain, and the expressions of *FASN*, *SREBP-1c*, *ChREBP* and *PP2A* was upregulated to different folds. After the termination of BCM treatment or inoculating with normal gut microflora, rats showed less epididymal fat pad and daily weight gain, and the expressions of these four genes were largely downregulated. These results might illustrate that the inhibition of colonic methanogens induced the increased expression of *SREBP-1c* and *ChREBP* through activating *PP2A*, and then caused the upregulation of *FASN*, which finally increased the body fat mass. Moreover, as we know that white adipose tissue is not only a major site of energy storage and important for energy homeostasis but it is also recognized as an important endocrine organ that secretes large numbers of biologically active ‘adipokines’ (Kadowaki *et al*., 2006). Of these adipokines, adiponectin has been widely focused for its anti-diabetic and anti-atherogenic effects and is expected to be a novel therapeutic tool for diabetes and the metabolic syndrome (Kadowaki and Yamauchi, 2005). In the present study, the expression of *adiponectin* was only detected in the epididymal fat pad of rats; BCM treatment significantly increased the expression of *adiponection* gene. We also found that the increased expression of *adiponectin* was accompanied with the upregulation of *PPARγ*, which is consistent with previous studies (Iwaki *et al*., 2003; Liu and Liu, 2012).

Leptin, which is mainly secreted by white adipose tissue, is an important signal in the regulation of adipose-tissue mass and body weight by inhibiting food intake and stimulating energy expenditure (Paracchini *et al*., 2005). Some reports suggested that leptin can directly induce the expression of *FASN* in adipose tissues (Huan *et al*., 2003). Researchers further conclude that low level of leptin might impart a signal to the microbiota to become more efficient at extracting calories from food (Bajzer and Seeley, 2006). In the current study, we found that the expression of *leptin* gene in individuals with methanogenic inhibition was not significantly affected. However, with the re-colonization of gut methanogens, the expression of *leptin* was upregulated in nearly all of the three tissues. In addition, we found that the up or downregulation of *leptin* was not accompanied with a consistent change of *FASN*, which is opposite to previous studies. This reason might be the complex regulation of *leptin*. Our results also suggested that there might be no direct connection between the expression of *leptin* and change of host's gut microbes.

In conclusion, with an SD rat model in the present study, BCM treatment significantly reduced the faecal methanogen populations with no effect on the bacterial community, and this effect was coupled with the change of fat metabolism in the rat as revealed by blood parameters and fat metabolism-related gene expression. Although the direct link between gut methanogen populations and energy metabolism needs further elucidation, our present approach using this SD rat model with inhibited colonic methanogens may provide new tool to investigate the effect of gut methanogen populations on the energy or adipose metabolism of healthy host.

## Experimental procedures

### Animal trial

A pre-experimental trial was conducted to determine the duration of the treatment with BCM to reduce the methanogen population in SD rats. A total of 18 8-week-old healthy male SD rats were divided into two groups (nine for each group), control and treatment. Rats in the treatment group were dosed daily with BCM (Supelco, USA) diluted to 0.3 mg per 100 kg of live body weight with sterilized saline solution by intragastric administration for 60 days, while control rats were dosed similarly with saline solution. Faecal sample of each rat was collected at 10-day intervals for quantification of methanogens.

Following the pre-experiment, a total of 45 8-week old healthy male SD rats (approximately 200 g live weight) were selected as experimental animals. They were randomly divided into five groups (nine rats for each group, one cage for each individual) including one control and four treatments. Rats in the treatment group were dosed with BCM as described above, while control rats were dosed similarly with saline solution. The experiment lasted 60 days with two separate 30-day experimental periods. At the end of the first period, all rats in the control and one of treatment groups were sacrificed. For the second period, the remaining three treatment groups were further used: one group continued with BCM treatment (CO), one stopped with BCM treatment (ST), while the other one was inoculated with 10 ml faecal fermentation liquids (incubated at 38°C for 48 h) from healthy rats (IN) on bedding to each cage to simulate the recolonization of gut methanogens. At the end of the second period, all rats were sacrificed.

The animals were fed a standard rodent diet (National Research Council) and raised at constant temperature (25°C) and humidity (70%) on a 12 h light/dark cycle. Rats were fasted (with free access to water) overnight. Food intake for each rat was recorded daily; each rat was weighed every week. Faecal samples were collected every 10 days and stored at −20°C for the microbial population analysis. When rats were sacrificed, blood was collected for biochemical analysis, and epididymal fat pad from each rat was removed from the body and weighed. Tissues of the colon, liver and epididymal fat of each rat were collected and stored at −70°C for gene expression analysis.

Both animal trials and sample collections were carried out at Small Animal Experimental Center of Nanjing Jinlin Hospital (Nanjing, China), and all procedures were approved by Nanjing Jinlin Hospital Animal Care and Use Committee.

### Serum biochemical analysis

Blood samples were collected and centrifuged at 2000 r.p.m./min for 10 min to collect serum. Supernatants were collected into new sterilized tube and stored at 4°C. An Olympus AU400 Biochemical Analyzer (Tokyo, Japan) was used to determine the concentration of HDL, LDL, cholesterol, glucose and triglyceride in blood serum.

### Real-time PCR and PCR-DGGE

Faecal samples collected at the end of each experimental period were used for deoxyribonucleic acid (DNA) extraction with the described repeated bead-beating method (Yu and Morrison, 2004). Bacteria and methanogen populations were quantified by real-time PCR on an Applied Biosystems 7300 Real-Time PCR System (Applied Biosystems, USA) using SybrGreen as the fluorescent dye. A reaction mixture (25 μl) consisted of 12.5 μl of IQ SYBR Green Supermix (Bio-Rad), 0.2 μM of each primer set and 5 μl of the template DNA. The amount of DNA in each sample was determined in triplicate, and the mean values were calculated. Standard curves were generated by using the serially diluted 16S rRNA or *mcrA* gene amplicons obtained from the respective target strains. Primer sets Bact1369/Prok1492, Bact934F/Bact1060R and Firm934F/Firm1060R were used for the quantification of total bacteria, Bacteroidetes and Firmicutes according to the description of previous studies (Suzuki *et al*., 2000; Guo *et al*., 2008). The copies of *mcrA* gene of methanogens were determined with primer pair qmcrA-F/qmcrA-R (Denman *et al*., 2007).

Primers U968-GC and L1401 were used to amplify the V6-V8 variable regions of the bacterial 16S rRNA gene (Nübel *et al*., 1996). Polymerase chain reaction amplicons obtained from V6-V8 regions of 16S rRNA genes were separated by DGGE using a DCode system (Bio-Rad, Hercules, CA, USA). Denaturing gradient gel electrophoresis was performed according to the specifications of Su *et al*. (Su *et al*., 2008). Denaturing gradient gel electrophoresis gels were scanned using GS-800 Calibrated Densitometer (Bio-Rad) and analysed using the software of Bionumerics 4.5 (Applied Maths, Kortrijk, Belgium).

### RNA extraction, cDNA synthesis and RT-PCR

Total RNA of the colon, liver and epididymal fat was isolated using TRIzol (Invitrogen, China), and 1 μg RNA was reverse transcribed with standard reagents (Biocolors, China). The reaction system was 10 μl including 5 μl SYBR, 1 μl DNA (100 ng μl^−1^), 0.5 μl forward and reserve primers (10 mM μl^−1^) and 3 μl double distilled water. The expression of *PPARγ*, *LPL*, *Fiaf*, *PP2A*, *SREBP-1c*, *ChREBP*, *FASN*, *leptin* and *adiponectin* mRNA was measured by quantitative real-time PCR with SybrGreen (Roche, Switzerland), and fluorescence was detected on an Applied Biosystems (ABI) 7300 sequence detector (He *et al*., 2004; Cong *et al*., 2007; Jun *et al*., 2008). Samples were incubated in the ABI 7300 sequence detector for an initial denaturation at 95°C for 10 min, followed by 35 PCR cycles of 95°C for 15 s, 60°C for 1 min and 72°C for 1 min. The expression of the genes was calculated relative to the expression of *GAPDH*, *β-actin* and *HPRT* with formula 2^-ΔΔCt^. Amplification of specific transcripts was confirmed by melting curve profiles at the end of each PCR.

### Statistical analysis

Statistical software Statistical Package for the Social Sciences (spss 17.0) was used for data statistics. Student's *t*-test was used to analyse the difference of data between the control and BCM treatment groups during the first experimental period. One-way analysis of variance (ANOVA) program was used for significance analysis of data from CO, ST and IN groups during the second experimental period. Significant differences were declared when *P* < 0.05.

## Supporting Information

**Table S1.** 16S rRNA gene copies of total bacteria, Firmicutes and Bacteroidetes in the feces of rats in the control and treatment groups.
