# Supplementary material for: Responses in gut microbiota and fat metabolism to a halogenated methane analogue in Sprague Dawley rats
Source: Microb Biotechnol. 2015 Mar 6;8(3):519–26. doi: 10.1111/1751-7915.12256 (PMC4408184; doi:10.1111/1751-7915.12256)
Supplement: Supplementary file 1 [file mbt20008-0519-sd1.doc]

Table S1 16S rRNA gene copies of total bacteria, Firmicutes and Bacteroidetes in the feces of rats in the control and treatment groups.

| Time (d) | Groups | Total bacteria | Firmicutes | Bacteroidetes |
| --- | --- | --- | --- | --- |
| 0 | Control | 10.25±0.19 | 10.11±0.14 | 9.47±0.26 |
|  | Treatment | 10.27±0.18 | 10.08±0.14 | 9.59±0.43 |
| 10 | Control | 10.61±0.35 | 10.42±0.13 | 10.07±0.34 |
|  | Treatment | 10.45±0.68 | 10.27±5.84 | 9.78±0.46 |
| 20 | Control | 10.71±0.22 | 10.47±0.06 | 10.05±0.03 |
|  | Treatment | 10.67±0.10 | 10.35±0.60 | 9.94±0.12 |
| 30 | Control | 10.60±0.25 | 10.29±0.55 | 9.85±0.15 |
|  | Treatment | 10.35±0.68 | 10.19±0.07 | 9.69±0.44 |

There was no significant difference between control and the treatment groups.
